# The possible role of the da Vinci robot for patients with vulval carcinoma undergoing inguinal lymph node dissection

**DOI:** 10.4274/jtgga.2016.0193

**Published:** 2017-06-01

**Authors:** Christos Iavazzo, Paraskevi-Evangelia Iavazzo, Ioannis D. Gkegkes

**Affiliations:** 1 Department of Gynecological Oncology, Northampton General Hospital, Northampton, United Kingdom; 2 Rural Practice, Molos Fthiotida, Greece; 3 First Department of Surgery, General Hospital of Attica “KAT”, Athens, Greece

**Keywords:** Da Vinci, vulval cancer, lymphadenectomy, robotics

## Abstract

Inguinal lymph node dissection represents the gold standard of treatment for patients with vulval carcinoma. The application of minimally invasive techniques, such as robotics, in the surgical treatment of gynecologic cancer, reduced the rate of postoperative complications, which has an important impact on the quality of patients’ life. Robotic inguinal lymph node dissection is a safe and oncologically effective but expensive and time-consuming approach in patients with penile cancer or melanoma. However, it is related with less postoperative complications, especially less lymphocele or lymphedema rates, and can improve the patients’ quality of life while minimizing cost for health systems. The introduction of robot- assisted inguinal lymph node dissection in the treatment of vulval carcinoma may be identified as a provisional option for the gynecologic oncologist. Our intention was to present a brief review/commentary on the possible use of a robot-assisted technique on inguinal lymphadenectomy for patients with vulval cancer.

## INTRODUCTION

The gold standard of vulval cancer management includes radical vulvectomy or wide local excision to achieve removal of the primary tumor followed by inguinal lymphadenectomy. Historically, Basset (1) in 1912 was the first to describe better survival rates of up to 74% in patients who had radical vulvectomy with an inguinal and pelvic lymphadenectomy (butterfly technique). Later, Taussig (2) used a less aggressive approach with separate incisions for the groin dissection and the vulvar excision with equally good results. However, such an approach was not widely accepted until 1981 when Hacker et al. (3) reported his study showing that the 5-year survival rate was 97%. Nowadays, the Royal College of Obstetricians and Gynecologists guidelines suggest wide local excision of the vulval tumor, followed by inguinal lymph node dissection performed using a triple-incision approach to minimize postoperative complications (4).

Inguinal lymph node dissection in women with vulval carcinoma is related with high postoperative complication rates e.g. lymphocyst, skin flap necrosis, and lymphedema or even up to 50% of wound infections (5). These morbidity rates are mainly related to the traditional open approach, and for this reason, some surgeons tried to develop minimally invasive techniques for inguinal lymph node dissection (6). Bishoff et al. (7) were the first to present a minimally invasive endoscopic approach for the groin node dissection. Josephson et al. (8) reported the first case of robotic inguinal lymphadenectomy. The main advantages of the robotic approach compared with laparoscopy include comfort for the surgeon, and a 3D approach with high magnification and instruments with a higher degree of freedom, especially in the limited working space of the groin (9).

The aim of our manuscript was to present the robotic approach as a provisional option for patients with vulval cancer undergoing groin node dissection. Our intention was to present a brief review/commentary on the possible use of a robot-assisted technique on inguinal lymphadenectomy for patients with vulval cancer. The technique, advantages, and surgical challenges are discussed based on similar experience of other surgical specialties (e.g. urology, plastic surgery).

### Technique

- Positioning the patient in a low lithotomy

- Bladder catheterization

- Surgical landmarks recognized

- Preparation of the surgical field with iodine solution

- Robot is located at 45° to the left side of the patient

- Assistant sits contralateral to the robot on the right side of the patient

- Femoral triangle is identified and a 2-cm incision is performed about 3 cm below its inferior aspect

- Scarpa’s fascia is identified and after blunt-finger dissection, the scope is used to create a superficial subcutaneous flap by sweeping the lens under the fascia

- Pneumoperitoneum up to 15-20 mm Hg pressure

- Three robotic trocars are used (two 8-mm and one 10-mm trocar)

- Bipolar Maryland and monopolar scissors are the main instruments used

- The boundaries of the dissection are similar to the open approach

- Surgical specimens removed in a laparoscopic bag

- Saphenofemoral junction is exposed after opening the fascia lata and deep pelvic lymph node dissection can also be performed if necessary

- Hemostasis checked

- Suction drains are placed bilaterally

- Trocar incisions are closed in standard fashion

## DISCUSSION

There is not enough evidence regarding the role of the da Vinci robot (Intuitive Surgical, Sunnyvale, USA) in groin node dissection. The approach has never been used for patients with vulval cancer; however, it has been used in patients with melanoma or penile cancer (6, 8, 10, 11, 12). We present the possible advantages of such an approach in patients with vulval cancer based on the experience from other specialties or the videoscopic technique (13, 14, 15). The evidence from videoscopic groin node dissections suggests that the postoperative complication rates are lower compared with the open approach. More specifically, Lu et al. (16) performed a laparoscopic groin node dissection in 15 patients with vulvar cancer. The authors showed that only one patient with diabetes developed a wound infection. The rate of skin-related postoperative complications was 20% and the rate of cellulitis was 10%, which was significantly lower compared with the open technique (16). Similarly, another study presented the results of patients with penile cancer who were treated robotically with no surgical site complications (6). Kharadjian et al. (11) revealed that none of the 26 patients who underwent robotic groin node dissection developed any lymphocele or lymphedema, which implies that the risk of developing such complications is minimized. This can obviously be related to improved postoperative quality of life and body image, while it minimizes the cost of postoperative care for health systems. However, such early findings with such short follow-up, albeit encouraging, should be further clarified with randomized controlled trials.

Current data from patients with melanoma or penile cancer who underwent robotic groin node dissection suggest that it is safe and oncologically effective, and the morbidity rates seem to be lower compared with open surgery. The above mentioned are supportive for the future use of minimally invasive techniques in groin node dissections in patients with vulval cancer. The main advantages of the procedure include lower postoperative morbidity, shorter hospital stay, less time for recovery, less postoperative pain, and better cosmetic appearance. Regarding the nodal yield of the new approach, two studies showed comparable results to the traditional open approach (7.1 vs. 7.2, respectively, with 1.6 vs. 1.8 positive nodes respectively) (8, 13). On the other hand, longer surgical time is essential for the minimally invasive approach, which can obviously increase the operation cost, but this could be improved in parallel to the learning curve of each individual surgeon.

Local recurrence rates after six years of follow-up can reach 6.6% in the robotic versus 7.7% in the open approach (13). Tobias-Machado et al. (17) showed that there was no local or systemic recurrence during a mean follow-up of 31.93 months in patients who underwent laparoscopic groin node dissection. For all the above reasons, Kharadjian et al. (11) concluded that the robotic method maintained consistency with oncologic principles in patients with penile cancer.

Several limitations can be found in such a newly developed approach. The retrospective nature of the available studies is one of them. Prospective randomized controlled trials are necessary to clarify the morbidity rates and advantages of this new approach. The main objections that could be raised include cost and learning curve; however, the long-term benefits of robotic inguinal lymph node dissection must be weighed against the cost of the da Vinci robot. Nevertheless, the robot-assisted technique could be proved cost-effective in centres with large series of patients (18). Moreover, the risk of port site metastasis should also be clarified in the future. Iavazzo and Gkegkes (19) showed that the port site metastasis rate in patients who had robotic operations for other gynecologic cancers was an extremely rare entity and was related to various factors, among which was the improper manipulation of the removed tumor. To date, no port site metastasis has been reported in the literature for patients who underwent robotic groin node dissection.

However, such a time-consuming minimally invasive approach in a closed field might be challenged by traditional surgeons. Gyneoncologists with expertise in robotic surgery and familiarity with groin node dissection should perform such operations in order to achieve similar oncologic outcomes to the traditional technique, including lymph node count and survival. Optimization of patient selection can further clarify the reported surgical outcomes such as operative time, nodal yield, morbidity, five-year survival, and recurrence rates. Naldini et al. (20) suggested that a multicenter prospective randomized study would clarify whether the minimally invasive approach for patients with vulval cancer could replace the standard open approach used for groin node dissection.

## CONCLUSION

By learning from other specialties’ experience, we can see that robotic groin dissection is safe and oncologically effective, but expensive and a time-consuming approach in patients with penile cancer or melanoma. The fact that it is related with fewer postoperative complications, especially less lymphocele or lymphedema, could lead to improvements in patients’ quality of life and minimize cost for health systems. For these reasons, in our opinion such an approach should also be applied in patients with vulval cancer, even though, in order to reach safe conclusions, randomized trials should be performed.
